# Combining External Medical Knowledge for Improving Obstetric Intelligent Diagnosis: Model Development and Validation

**DOI:** 10.2196/25304

**Published:** 2021-05-10

**Authors:** Kunli Zhang, Linkun Cai, Yu Song, Tao Liu, Yueshu Zhao

**Affiliations:** 1 School of Information Engineering Zhengzhou University Zhengzhou China; 2 The Third Affiliated Hospital of Zhengzhou University Zhengzhou China

**Keywords:** intelligent diagnosis, obstetric electronic medical record, medical knowledge, attention mechanism

## Abstract

**Background:**

Data-driven medical health information processing has become a new development trend in obstetrics. Electronic medical records (EMRs) are the basis of evidence-based medicine and an important information source for intelligent diagnosis. To obtain diagnostic results, doctors combine clinical experience and medical knowledge in their diagnosis process. External medical knowledge provides strong support for diagnosis. Therefore, it is worth studying how to make full use of EMRs and medical knowledge in intelligent diagnosis.

**Objective:**

This study aims to improve the performance of intelligent diagnosis in EMRs by combining medical knowledge.

**Methods:**

As an EMR usually contains multiple types of diagnostic results, the intelligent diagnosis can be treated as a multilabel classification task. We propose a novel neural network knowledge-aware hierarchical diagnosis model (KHDM) in which Chinese obstetric EMRs and external medical knowledge can be synchronously and effectively used for intelligent diagnostics. In KHDM, EMRs and external knowledge documents are integrated by the attention mechanism contained in the hierarchical deep learning framework. In this way, we enrich the language model with curated knowledge documents, combining the advantages of both to make a knowledge-aware diagnosis.

**Results:**

We evaluate our model on a real-world Chinese obstetric EMR dataset and showed that KHDM achieves an accuracy of 0.8929, which exceeds that of the most advanced classification benchmark methods. We also verified the model’s interpretability advantage.

**Conclusions:**

In this paper, an improved model combining medical knowledge and an attention mechanism is proposed, based on the problem of diversity of diagnostic results in Chinese EMRs. KHDM can effectively integrate domain knowledge to greatly improve the accuracy of diagnosis.

## Introduction

Intelligent diagnosis is a way to provide clinical decision support for doctors by means of artificial intelligence technology. In the clinic, intelligent diagnosis plays an important role and can be applied to a variety of practical situations. Intelligent diagnosis can help doctors diagnose a patient’s condition, significantly improving the efficiency and accuracy of the diagnosis, and the results can also become an important basis for future diagnosis. The continuous development of modern diagnosis and treatment technology has made medical information increasingly complex. Doctors obtain a large amount of clinical diagnostic information every day and need to make comprehensive decisions based on a large amount of data representing clinical information [1]. In addition, the occurrence of complications during pregnancy poses a challenge to doctors.

Electronic medical records (EMRs) are the most detailed and direct form of clinical medical activities [2]. With the rapid growth of EMRs, many methods of intelligent diagnosis using EMRs have become available, enabling significant progress in this field. Early intelligent diagnosis works mainly relied on artificially designed feature templates [3,4] or used single traditional machine learning methods, treating intelligent diagnosis as a classification problem. Goldstein et al [5] used the Informatics for Integrating Biology & the Bedside 2008 dataset to train a classifier for each disease category to classify obesity and 15 other complications. Medhekar et al [6] developed a decision support system based on data mining that used a naïve Bayes classifier to model heart disease. Roopa et al [7] used principal component analysis to extract the characteristics of a diabetes dataset and then used a linear regression model to predict whether a patient had diabetes. These methods promoted the application of machine learning and natural language processing in intelligent diagnosis but are still in the early stages (eg, using relatively simple classification methods and a shallow analysis of the EMRs).

Recently, an increasing number of researchers have focused on neural networks to model intelligent diagnosis and related tasks. Yang et al [8] proposed a clinical assistant diagnosis method based on a multilayer convolutional neural network [9]. This method uses self-learning to automatically extract the high-level semantic information from EMRs. Chen et al [10] used an end-to-end hierarchical neural network to investigate breast cancer problems using EMRs. Hao et al [11] used a deep belief network [12] to integrate patients’ structured data characteristics to predict the risk of cerebral infarction. Hao et al [13] proposed a diagnostic modeling and reasoning system using the dynamic uncertain causality graph and improved the diagnostic accuracy of jaundice. Jeddi et al [14] applied the C5.0 algorithm to draw a multibranch decision tree used to aid in the diagnosis of complicated skin diseases.

When the scale of the training data is limited in a traditional neural network, the advantage of using external knowledge is more obvious. These methods ignore the fact that neural networks and external knowledge can benefit from each other.

The rapid development of computer technology and biotechnology has enabled the rapid growth of biomedical text resources. These resources contain valuable knowledge that can be used to promote the development of medical informatics. A doctor’s diagnostic process is a combination of their own clinical experience and general medical knowledge. Therefore, medical knowledge is indispensable in the diagnosis process. Fang et al [15] proposed a method to diagnose chronic obstructive pulmonary disease based on a knowledge graph and integrated models. Liang et al [1] designed a system framework for the data mining of EMRs based on pediatric diseases. This framework combines medical knowledge with a data-driven model and uses logistic regression for the disease hierarchical diagnosis. These efforts provide new methods for medical data analysis, but intelligent diagnosis based on EMRs is still hindered by the following problems:

- An EMR usually involves multiple diagnostic results, such as normal diagnosis, pathological diagnosis, and complications.
- In the aspect of external knowledge, the above methods simply splice the knowledge with the model, which fails to capture the key information well and requires a large number of calculations.
- To achieve the most advanced performance, doctors not only care about the diagnostic results but also need to know what medical knowledge contributed to the diagnosis.

Therefore, in this paper, we design a novel intelligent diagnosis model based on deep learning. Specifically, to capture the important details of the original documents, we use bidirectional gated recurrent units (Bi-GRUs) [16] with a hierarchical attention mechanism to model the correlations among words and sentences in EMRs and knowledge documents. Given an analysis of the correlation between the EMRs and medical knowledge documents, we select the most supportive external knowledge to support intelligent diagnosis. Considering the diversity of diagnostic results, we need to conduct intelligent diagnosis in the multilabel classification paradigm. The major contributions of this paper are summarized as follows:

- Knowledge-aware hierarchical diagnosis model (KHDM) makes full use of the hierarchical deep language model to encode the EMRs and external knowledge documents.
- Language model is enriched with high-quality knowledge, combining the advantages of both to perform a knowledge-aware diagnosis.
- Experimental results on real-word Chinese obstetric EMRs achieve superior performance over baselines. In addition, we discuss the importance and interpretability of external medical knowledge.

## Methods

### Overview

**Figure 1:**
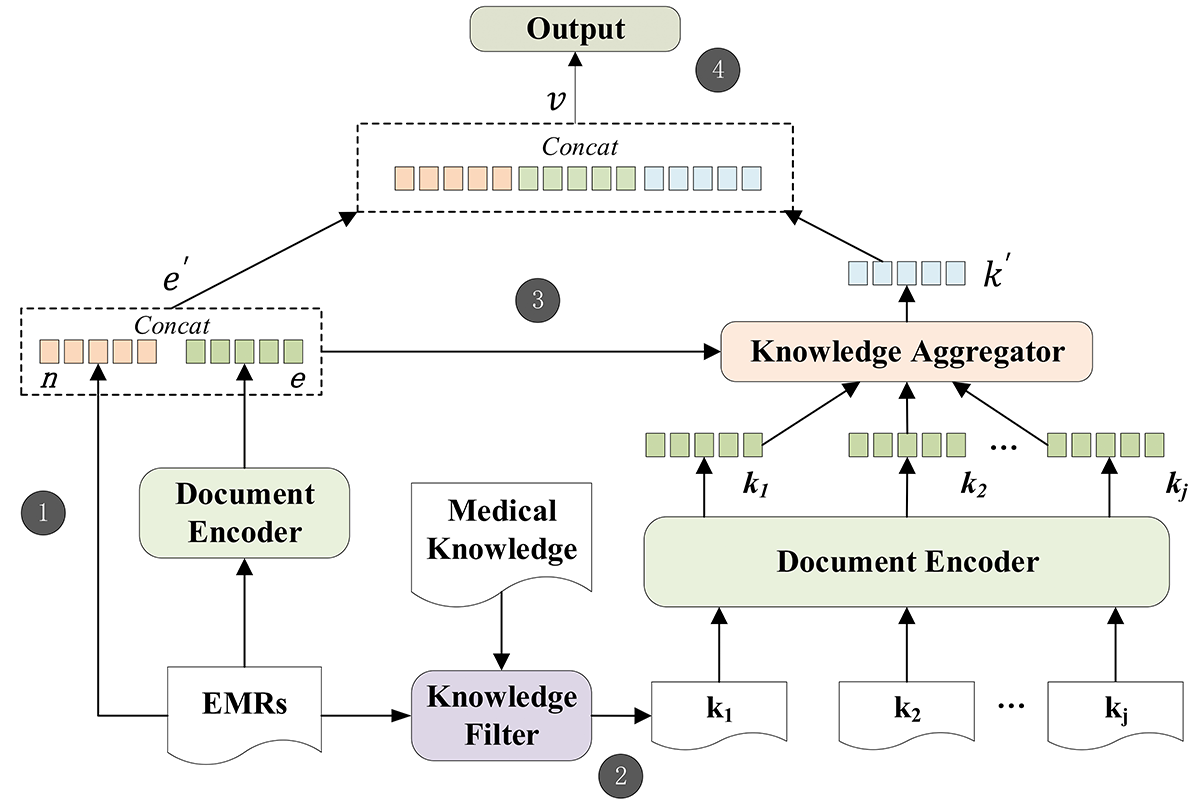
Overview of knowledge-aware hierarchical diagnosis model.

KHDM contains the following steps, as depicted in Figure 1.

1. Enter the EMR into the document encoder to obtain the document embedding *e* and concatenate it with the numerical features *n* to get the final EMR embedding *e′*.
2. Input the EMRs and external knowledge documents into the knowledge filter for preliminary screening of the external knowledge, and send the filtered knowledge documents to the document encoder to obtain the knowledge embedding *k*.
3. Input the EMR embedding and knowledge embedding jointly into the knowledge aggregator. Through the simultaneous analysis of the EMRs and knowledge documents, our model learns a knowledge-side attention component in order to carefully select the most supportive knowledge document *k′* from the external knowledge to support intelligent diagnosis.
4. *e′* and *k′* are concatenated and passed to a sigmoid classifier for the diagnosis. In this section, we introduce the document encoder, knowledge attention module (including the knowledge filter and knowledge aggregator), and output.

### Document Encoder

The purpose of the document encoder is to encode the original EMRs and knowledge documents into continuous low-dimensional embeddings to capture semantic relationships. EMRs and medical knowledge documents usually have potential hierarchical structures. A document consists of several sentences, and a sentence consists of several words. Intuitively, the document embedding problem can be converted into two sequence embedding problems [17]. Modeling the semantics of the EMR and external knowledge by word-level and sentence-level representations can fully capture the hierarchical laws and dependencies.

The words and sentences in a document provide different information and have different degrees of importance. Inspired by Yang et al [18], we successively apply the attention mechanism [19] at the word level and sentence level so that it can differentiate more important information when constructing the document representation. The attention mechanism not only improves the performance of the deep learning model but also intuitively shows the contributions of words and sentences to the classification decision.

We use the Bi-GRU sequence encoder with an attention mechanism to encode the EMRs and knowledge documents. Numerical features, such as physiological indicators and laboratory results, are also important in EMRs. To enable more complete use of the EMRs, we separately extract the numerical features and concatenate them with EMRs. Next, we introduce the Bi-GRU sequence encoder, attention encoder, and numerical features in detail.

**Figure 2:**
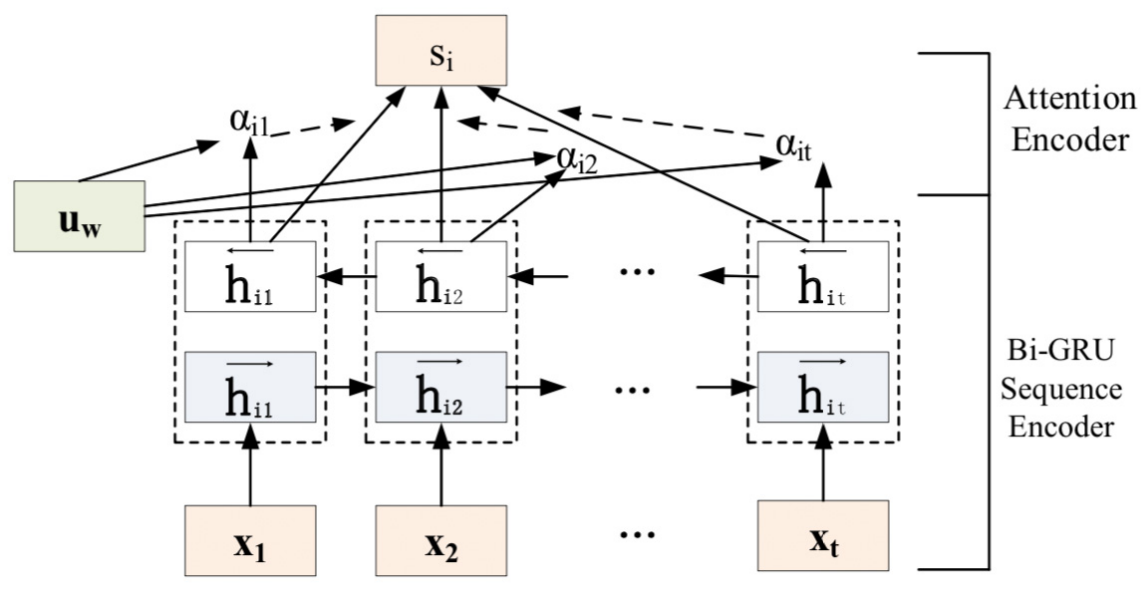
Document encoder framework.

Although the word-level and sentence-level encoders can have different structures, we use the same structure here for simplicity, as shown in Figure 2.

### Bi-GRU Sequence Encoder

The importance of words and sentences is highly context dependent. In other words, the same words or sentences may have different degrees of importance in different contexts. We model the semantics of EMRs and external knowledge documents by including word-level and sentence-level representations that can fully capture hierarchical dependencies. Taking the word level as an example, we use Bi-GRU to make a word compilation of the meaning of an entire sentence, where the GRU uses a gate control mechanism to memorize the information of the previous cells.

The GRU has two gates: the reset gate *r_t_* and the update gate *z_t_*. The reset gate is used to determine the degree to which the previous information is forgotten, and the update gate is used to decide which information to forget and which new information to enter. *r_t_* and *z_t_* jointly control the calculation from hidden state *h_t_*_–1_ to hidden state *h_t_*. *h_t_~* is a candidate hidden layer. At time t, the GRU is calculated as follows:


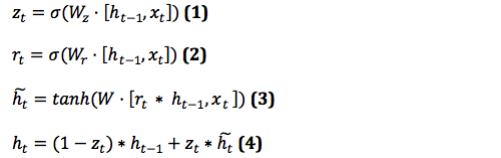


where W_*_ is the weight matrix. *x_t_* is the sequence vector at time t, and σ is the activation sigmoid function that converts the values of each cell state into the range of 0 to 1 to act as a gate signal. The reset gate *r_t_* receives the values of *h_t_*_–1_ and *x_t_*. If *r_t_* is zero, then the previous state is not saved. In other words, at this time, *h_t_ ~* only contains the information of the current word. Afterward, the update gate *z_t_* controls how much information needs to be forgotten from the hidden state *h_t_*_–1_ at the previous moment and how much hidden layer information *h_t_ ~* needs to be added at the moment. The final hidden layer information *h_t_* can then be output.

Bi-GRU uses forward and backward GRUs to encode the sequence in two directions so that the associations between different words (sentences) are taken into account when encoding. Specifically, consider an EMR e = [s_1_,s_2_,^...^,s_L_], where L is the number of sentences and s_i_(1 ≤ *I* ≤ *L*) represents the *i^th^* sentence in the document. For each sentence in the document s_i_ = [w_i1_,w_i2_,^...^,w_iT_], w_im_(1 ≤ m ≤ T) represents the *m^th^* word in s_i_. w_im_ is the embedding representation of w_im_, and the encoding method is to concatenate the feature representations of Bi-GRU; that is, the forward hidden state *h_it_*^→^ and backward hidden state *h_it_*^←^ at time t are weighted sums:


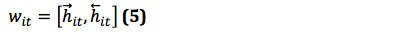


### Attention Encoder

Not all words have the same effect on the meaning of a sentence, as is the case for sentences within documents. The attention mechanism has become an effective mechanism for mining local differences and highlighting vital elements of data. Therefore, we add an attention mechanism at the word and sentence levels to indicate their importance to the previous level. Compared with the general word-level attention mechanism, the sentence-level attention mechanism plays a more important role in medical documents because certain domain phrases often appear. At the word level, the attention mechanism is introduced to extract those words that are important to the meaning of the sentence, and the representations of these informative words are aggregated to form a sentence vector. The final sentence vector representation *s_i_* is defined as follows:


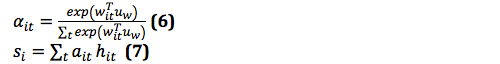


where the weight *a_it_* indicates the importance of a word to the meaning of the sentence. The context vector *u_w_* is an attention matrix obtained by a random initialization method. It is a cumulative sum of the different probability weights assigned by the attention mechanism and the performance of each hidden layer state. We measure the importance of the word as similarity of *w_it_* with a word-level context vector *u_w_* and get a normalized importance weight *α_it_* through a softmax function. We use the same method to obtain the context-level representation of *u_s_* and finally to obtain the document vector *e*:


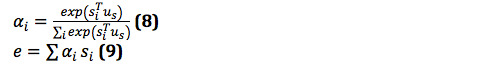


### Numerical Features

Numerical features are very important indicators in Chinese obstetric EMRs. For example, physiological indicators such as the age of the pregnant woman, the number of menopause months, and the uterine height are important factors affecting the clinical judgement. However, there are some cases where the numerical units of EMRs are not uniform. Taking the number of menopause months as an example, it is generally described as “menopause X months,” but some EMRs also use the description method “menopause Y weeks,” We unified the units of this indicator as months, relying on the equation that “4 weeks” is approximately “1 month” in the feature extraction. We also need to consider the validity of the data. According to medical professional knowledge, numerical features have a certain value range. For example, when extracting the physiological parameters of a pregnant woman’s uterine height, if a value is found to be “29 m,” it can be speculated that this data point is incorrect, which will affect the experimental results. This paper determines the accuracy of the data by setting thresholds for each physiological index, and the error data are directly deleted. Detailed thresholds descriptions are provided in Multimedia Appendix 1. After extracting the numerical features *n*, they are concatenated with the document vector *e* as the final representation of the EMR:


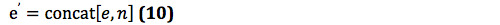


### Knowledge Attention Module

Integrating all the external knowledge into the model is very time-consuming, and not all knowledge has enough discernment to support the final classification. Our knowledge attention module aims to alleviate these problems, ensuring that our model can select reliable and useful knowledge for each candidate. This module consists of a knowledge filter and knowledge aggregator. The knowledge filter can preliminarily filter out irrelevant knowledge documents, and the knowledge aggregator uses the attention mechanism to select the most supported knowledge. Considering that external knowledge has too much noise, such an attention mechanism explores the correlation between the EMRs and knowledge documents. KHDM mainly uses this module to make a knowledge-aware diagnosis.

#### Knowledge Filter

We consider the task of the knowledge filter to be text similarity calculation. By calculating the similarity between the input EMRs and the medical knowledge documents, the knowledge not related to the input EMRs will be filtered out. Due to the special nature of medical texts, symptoms and diagnostic methods vary by disease. Therefore, we use the term frequency–inverse document frequency (TF-IDF) to extract the text features of the EMRs and external knowledge. TF(x) represents word frequency, which counts the frequency of each word in an EMR. IDF(x) represents the inverse text frequency and returns the frequency of word x in the corpus, reflecting the importance of words in the text:


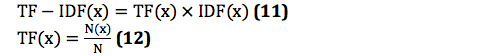


where N(x) represents the number of occurrences of word x in the document, N is the total number of words in the document, and D is the total number of documents. D(x) indicates how many documents the word x appears in. Due to professionalism in the medical field, the IDF is smoothed so that domain words that do not appear in all documents can also obtain a suitable IDF value:


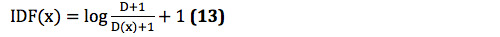


The set of documents and knowledge is then viewed as a set of vectors in a vector space. The cosine function is used to measure the similarity between the document and any knowledge. If the similarity score is less than 0.5, we consider these knowledge documents irrelevant and vice versa. After that, we use the document encoder mentioned above to encode the relevant knowledge document. Finally, we obtain the relevant knowledge vector representation: *k* = [*k*_1_,*k*_2_,^...^,*k*_j_].

#### Knowledge Aggregator

This submodule aims to find further medical knowledge that supports intelligent diagnosis and generates an aggregated knowledge embedding *k*′. Therefore, we use the attention mechanism to select the key knowledge documents that are the most critical to the task objective. When generating an aggregated knowledge embedding, more attention is paid to the most important knowledge:


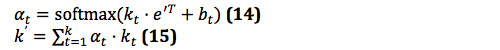


The attention weight *α_t_* generated by *k_t_* and *e*′ can be regarded as the correlation between the external knowledge and the input EMRs. The top *k*-related knowledge is selected according to the attention weight after sorting. The number of related knowledge documents less than *k* will be padded with zero vectors. We define *k* as the average label number per document.

### Output

To make the final diagnosis prediction, we first concatenate the EMR embedding *e*′ and the knowledge embedding *k*′ and feed it into two fully connected layers to generate a new vector, which is then passed to a sigmoid classifier to produce the predicted results. We consider that all diseases with an output probability greater than τ are positive predictions. The input to the first fully connected layer can also be only *e*′ or *k*′, which means we use only EMRs or external knowledge to make the diagnosis. The loss function for the training is the cross entropy:


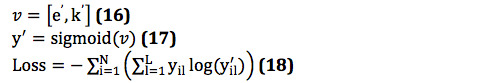


## Results

### Dataset Details

**Figure 3:**
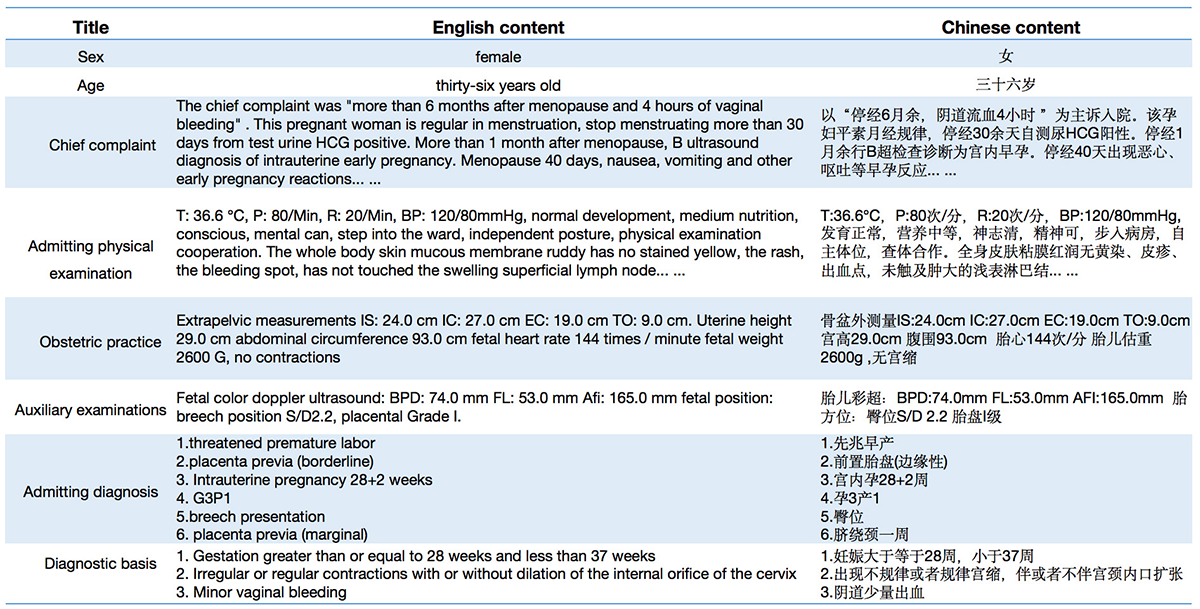
Chinese obstetric electronic medical record sample.

We collected 24,192 Chinese obstetric EMRs randomly selected by multiple hospitals as the research material, and each EMR corresponds to one patient. Due to the different writing habits of doctors, there are many different forms of expression for the same diagnostic results. Therefore, the medical thesaurus *International Classification of Diseases, Tenth Revision* [20] is used as the basis for the standardization of disease naming. To protect the privacy of patients, personal identifying information such names and ID numbers of patients was removed [21]. The dataset focuses on inpatient department data and consists primarily of structured and unstructured text data. Structured data include the basic information on the patient such as age, ethnicity, and laboratory examination data. Unstructured data mainly refer to the patient’s main complaint, admission, and physical examination. Detailed data descriptions are shown in Figure 3. The dataset contains 59 types of disease diagnostic results and is divided into 21,772 training sets and 2420 test sets according to the results distribution.

For external knowledge, we collected descriptions of medical concepts from the authoritative textbook *Obstetrics and Gynecology* [22] and a medical encyclopedia. The medical concepts mainly include the disease definition, symptoms, and treatment methods. In the end, we collect a total of 72 medical definition documents that make up our external knowledge. All external knowledge was chosen under the guidance of medical experts.

### Hyperparameter Setting

Since all EMRs and external knowledge documents are written in Chinese, we first use PKUSEG [23] to segment the document and set the maximum document length to 1600 characters. We use the GloVe [24] model to train word embedding on the corpus of EMRs after word segmentation. The hidden state size of the GRU is set to 100. For text convolutional neural network (TextCNN), this paper sets the filter width to (2, 3, 4, 5), and each filter size is 25 to maintain consistency. After the connection, the representation size of our model becomes 200. Finally, a 200 * *c* fully connected layer is added (*c* is the number of labels).

Since we use the sigmoid function for classification, the prediction threshold τ is set to 0.5. Average label number per document *k* is 2.688, so we set *k* = 3. We use Adam [25] as the optimizer. During the training period, EMRs are selected by random sampling method. We set the learning rate to 0.001 and the batch size to 32.

### Performance on an Obstetric EMR Dataset

In multilabel learning, each sample may have multiple category labels. Many evaluation metrics for multilabel learning have been proposed [26]. We use the average precision, 1-error, hamming loss, ranking loss, and coverage as evaluation metrics. The following text classification models were used as baselines for comparison:

- Classifier chains [27] integrate multiple single classification methods into one model to solve the problem of multilabel classification.
- Multilabel k–nearest neighbor [28] considers the k instances with the smallest distance from the new instance in the feature space as a set.
- Long short-term memory (LSTM) [29] uses the last hidden state as the representation of the whole document.
- Bidirectional long short-term memory (Bi-LSTM) is a bidirectional LSTM that can obtain long-term context information in the direction of the input.
- TextCNN [9] uses multiple kernels of different sizes to extract the key information in sentences to better capture the local relevance.

All text classification models are trained in the multilabel framework. The experimental results on the Chinese obstetric EMR dataset are summarized in Table 1.

**Table 1:** Comparative results on Chinese obstetric electronic medical record dataset.

Method	Average precision	1-error	Hamming loss	Ranking loss	Coverage
CC^a^	0.5083	0.4880	0.0308	0.1366	19.7917
ML-KNN^b^	0.6109	0.2488	0.0258	0.0709	10.2347
LSTM^c^	0.8651	0.0836	0.0166	0.0190	4.4612
Bi-LSTM^d^	0.8721	0.0775	0.0164	0.0186	4.4625
TextCNN^e^	0.8652	0.0961	0.0188	0.0203	4.6035
KHDM^f^	0.8929	0.0713	0.0156	0.0165	4.0833

^a^CC: classifier chains.

^b^ML-KNN: multilabel k–nearest neighbor.

^c^LSTM: long short-term memory.

^d^Bi-LSTM: bidirectional long short-term memory.

^e^TextCNN: text convolutional neural networks.

^f^KHDM: knowledge-aware hierarchical diagnosis model.

According to the experimental results, compared with the traditional machine learning methods, the neural network method has achieved better results. The main reason is that the neural network can capture richer features and deeper semantic information. Considering the structured context information, a bidirectional network can significantly improve the performance. For example, Bi-LSTM gives an average precision of 0.8721, while that of the LSTM is 0.8651. In addition, our model is largely superior to other traditional neural network methods. The TextCNN is usually connected to the pooling layer after the convolution layer. Its operation logic is to retain the strongest features from the feature vectors obtained from a convolution kernel so it cannot retain the relative position information of the original input, resulting in information loss. LSTM has a sequence dependency problem and does not perform well when the document is too long. Our model uses a hierarchical structure to divide the document into sentences without the problems of distance dependence and information loss. In general, our model is much better than the other models in all of the evaluation metrics applied, with improvements of 3% to 30%. Making full use of the attention mechanism to integrate external medical knowledge is undoubtedly an important way to improve the effectiveness of intelligent diagnosis.

### Performance on Public Dataset

This paper takes the obstetric intelligent diagnosis problem into a multilabel classification framework. Therefore, we test the classification effect on two public datasets: DeliciousMIL [30] and Hep categories. The former consists of a number of tagged pages on the social bookmarking site delicious.com, with categories including programming, style, and reference, and the latter is a public multilabel dataset available on Magpie, with subject categories relevant to high-energy physics (HEP) abstracts, including astrophysics, experiment-HEP, gravitation and cosmology, phenomenology-HEP, and theory-HEP. Table 2 provides a brief description of each dataset. The selected external knowledge *k* values of the two datasets are 3 and 1, respectively.

The external knowledge data for the DeliciousMIL and Hep categories datasets are derived from Wikipedia entry definitions. Table 3 and Table 4 present the results. Similar to the results on the obstetric EMR dataset, it can be clearly observed that our model performs best in multilabel text classification, proving that KHDM is universal for text classification tasks.

**Table 2:** Description of public datasets.

Dataset	Field	Instances	Labels	AL^a^
DeliciousMIL	Social networking sites	12,234	20	2.9574
Hep categories	High-energy physics	1000	5	1.1920

^a^AL: average label number per document.

**Table 3:** Comparative results on public dataset DeliciousMIL.

Method	Average precision	1-error	Hamming loss	Ranking loss	Coverage
CC^a^	0.3208	0.8134	0.2054	0.4183	12.9241
ML-KNN^b^	0.3703	0.7621	0.4748	0.3488	11.0213
LSTM^c^	0.5813	0.3947	0.1641	0.1518	6.9928
Bi-LSTM^d^	0.5968	0.3786	0.1610	0.1615	6.9648
TextCNN^e^	0.6299	0.3639	0.1760	0.1344	6.0637
KHDM^f^	0.6386	0.3312	0.1255	0.1284	5.9101

^a^CC: classifier chains.

^b^ML-KNN: multilabel k–nearest neighbor.

^c^LSTM: long short-term memory.

^d^Bi-LSTM: bidirectional long short-term memory.

^e^TextCNN: text convolutional neural networks.

^f^KHDM: knowledge-aware hierarchical diagnosis model.

**Table 4:** Comparative results on public dataset Hep categories.

Method	Average precision	1-error	Hamming loss	Ranking loss	Coverage
CC^a^	0.5606	0.6290	0.2982	0.4381	1.9410
ML-KNN^b^	0.5733	0.5800	0.3460	0.4433	2.2300
LSTM^c^	0.6807	0.5422	0.2740	0.2437	0.9642
Bi-LSTM^d^	0.7055	0.4816	0.2200	0.2251	0.9455
TextCNN^e^	0.7903	0.3429	0.2420	0.1550	0.6207
KHDM^f^	0.8929	0.0713	0.0156	0.0165	4.0833

^a^CC: classifier chains.

^b^ML-KNN: multilabel k–nearest neighbor.

^c^LSTM: long short-term memory.

^d^Bi-LSTM: bidirectional long short-term memory.

^e^TextCNN: text convolutional neural networks.

^f^KHDM: knowledge-aware hierarchical diagnosis model.

## Discussion

### Ablation Test

KHDM is a combination of a knowledge attention mechanism and external medical knowledge representation. We conducted an ablation test to assess the contributions of these two components in our model. Table 5 presents the performance of our model and its ablations on the obstetric EMR dataset. *w/o Knowledge* means using only the EMRs for the intelligent diagnosis, and *w/o Att* means we remove the attention mechanism and all the medical knowledge documents directly concatenated with the EMRs and do not use the knowledge attention module.

**Table 5:** Results of the ablation test.

Method	Average precision	One error	Hamming loss	Ranking loss	Coverage
w/o Knowledge	0.8789	0.1047	0.0184	0.0212	4.2364
w/o Att^a^	0.8519	0.1022	0.0164	0.0181	4.3210
TextCNN^b^	0.8652	0.0961	0.0188	0.0203	4.6035
TextCNN + knowledge	0.8700	0.0912	0.0167	0.0199	4.3516
KHDM^c^	0.8929	0.0713	0.0156	0.0165	4.0833

^a^Att: attention.

^b^TextCNN: text convolutional neural networks.

^c^KHDM: knowledge-aware hierarchical diagnosis model.

From the experimental results, the following can be seen:

- When the external knowledge is not introduced or the attention mechanism is not used, the model performance deteriorates.
- The models incorporating knowledge are superior to ordinary text classification models with a drop to 0.8789 of model *w/o Knowledge* after the supplementary knowledge is removed. The effectiveness of using external knowledge information is confirmed, and medical knowledge contributes to intelligent diagnosis.
- When fusing the medical knowledge, performances of .*w/o Att* and *TextCNN + knowledge* significantly increase by simply concatenating the knowledge document.

However, these models do not use the knowledge attention mechanism but directly concatenate with the external knowledge, which will introduce a large amount of noise. We can see KHDM improves more than 2 percentage points on most evaluation metrics. These ablation test results reflect the importance and rationality of using the attention mechanism to capture the interactions between multiple inputs.

### Interpretability of the Attention Mechanism

Interpretability is very important for model evaluation, especially in the medical field, as it allows doctors to understand the rationale behind the diagnostic results. To verify that our model can capture the most important sentences and words in a document, we first visualized the hierarchical attention mechanism in the document encoder on the Chinese obstetric EMR dataset.

**Figure 4:**
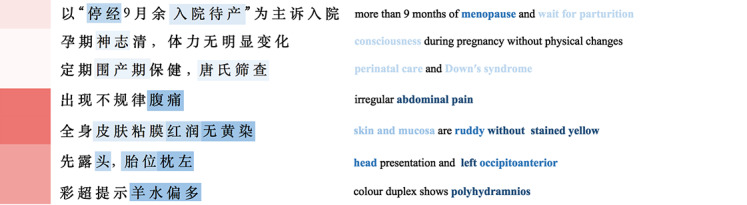
Visualization of attention in document encoder (attention encoder).

As shown in Figure 4, every line is a sentence, and we normalize the sentence weights and word weights to ensure that only the important words in the most important sentences are emphasized. Red denotes the weight of a sentence and blue denotes the weight of a word, where the darker the color is, the greater the weight. We know that doctors often diagnose patients by analyzing their clinical symptoms and test results. Our model accurately locates the words *abdominal pain* and *no yellow stain* and their corresponding sentences.

**Figure 5:**
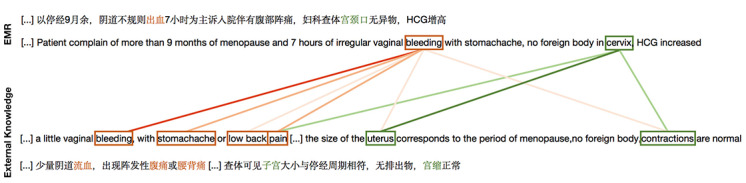
Visualization of attention in knowledge aggregator (knowledge attention).

Next, we choose a representative example to illustrate the role of the attention mechanism in the knowledge aggregator. We remove all attention values less than 10^–3^ from the visualization. As can be seen in Figure 5, our model pays more attention to the clinical symptom *blood (red part)* and site *cervix (green part)* within the medical knowledge. The darker the color of the line, the higher the attention. Similarly, medical concepts are essential in clinical diagnosis, so medical knowledge with a higher attention score through localization of symptoms and sites will be selected.

### Limitations

We used only external medical knowledge related to obstetric diseases, but obstetric diagnosis also involves immunology, cytology, genetics, pathology, and other multilevel knowledge. For cardiovascular and cerebrovascular diseases requiring blood pressure and routine blood tests, the numerical features are very important for the diagnosis, and our proposed method provide support. These numerical features are very important for the diagnosis, and our proposed method can provide support. But for diseases such as cancer, text data alone is not enough and must be combined with other types of medical information such as medical images and signals. To improve the interpretability of intelligent diagnosis model, communication with the clinic and selection of an appropriate interpretation method in terms of complementing the doctor’s workflow and habits is still necessary. Another limitation that needs to be addressed in achieving intelligent diagnosis based on EMRs is imbalanced datasets. This paper selects common diseases as the research object. In future work, we will focus on diseases with lower frequency.

### Conclusions

In this paper, we propose KHDM that synchronously and effectively uses Chinese obstetric EMRs and external knowledge. Particularly, the use of the knowledge attention module to selectively leverage medical knowledge not only improves performance but also provides a basis for intelligent diagnosis. The experimental results on a real obstetric EMR dataset show that KHDM can effectively use external knowledge to enhance the language model, thereby improving the performance.

## Appendix

Multimedia Appendix 1 Threshold descriptions in numerical features.

## Abbreviations

Bi-GRU: bidirectional gated recurrent unit
Bi-LSTM: bidirectional long short-term memory
EMR: electronic medical record
HEP: high-energy physics
KHDM: knowledge-aware hierarchical diagnosis model
LSTM: long short-term memory
TextCNN: text convolutional neural network
TF-IDF: term frequency–inverse document frequency
