# Supplementary material for: Combining External Medical Knowledge for Improving Obstetric Intelligent Diagnosis: Model Development and Validation
Source: JMIR Med Inform. 2021 May 10;9(5):e25304. doi: 10.2196/25304 (PMC8145091; doi:10.2196/25304)
Supplement: Multimedia Appendix 1 [file medinform_v9i5e25304_app1.docx]

| **Feature name** | **Normal range** | **Unit** |
| --- | --- | --- |
| Age | 0-100 | age |
| Temperature (T) | 35-41 | ℃ |
| Pulse (P) | 40-150 | Times/min |
| Respiratory (R) | 0-30 | Times/min |
| Systolic Pressure | 40-200 | mmHg |
| Diastolic Pressure | 0-200 | mmHg |
| Fundal Height | 0-50 | cm |
| Abdominal Circumference | 60-120 | cm |
| Transverse Outlet (TO) | 7-11 | cm |
| Menopause Time | 0-45 | week |
| External Conjugate (EC) | 0-40 | cm |
| Interspinal Diameter (IS) | 10-40 | cm |
| Intercristal Diameter (IC) | 0-40 | cm |
| Estimated Fetal Weight (EFW) | 0-7000 | g |
| Cardiac | 0-200 | Times/min |
| Femur Length (FL) | 35-100 | mm |
| Amniotic Fluid Index (AFI) | 0-500 | mm |
| Amniotic Fluid Volume (AFV) | 0-100 | mm |
| Hemoglobin (Hb) | 100-130 | g/L |
